# Computational Methods for Continuous Eye-Tracking Perimetry Based on Spatio-Temporal Integration and a Deep Recurrent Neural Network

**DOI:** 10.3389/fnins.2021.650540

**Published:** 2021-04-29

**Authors:** Alessandro Grillini, Alex Hernández-García, Remco J. Renken, Giorgia Demaria, Frans W. Cornelissen

**Affiliations:** ^1^Laboratory for Experimental Ophthalmology, University Medical Center Groningen, Groningen, Netherlands; ^2^Osnabrück University, Osnabrück, Germany; ^3^Cognitive Neuroscience Center, Department of Biomedical Sciences of Cells and Systems, University Medical Center Groningen, Groningen, Netherlands

**Keywords:** eyetracking algorithms, perimetry, continuous psychophysics, recurrent neural networks, threshold free cluster enhancement, computational method, eyetracking, glaucoma

## Abstract

The measurement of retinal sensitivity at different visual field locations–perimetry–is a fundamental procedure in ophthalmology. The most common technique for this scope, the Standard Automated Perimetry, suffers from several issues that make it less suitable to test specific clinical populations: it can be tedious, it requires motor manual feedback, and requires from the patient high levels of compliance. Previous studies attempted to create user-friendlier alternatives to Standard Automated Perimetry by employing eye movements reaction times as a substitute for manual responses while keeping the fixed-grid stimuli presentation typical of Standard Automated Perimetry. This approach, however, does not take advantage of the high spatial and temporal resolution enabled by the use of eye-tracking. In this study, we introduce a novel eye-tracking method to perform high-resolution perimetry. This method is based on the continuous gaze-tracking of a stimulus moving along a pseudo-random walk interleaved with saccadic jumps. We then propose two computational methods to obtain visual field maps from the continuous gaze-tracking data: the first is based on the spatio-temporal integration of ocular positional deviations using the threshold free cluster enhancement (TFCE) algorithm; the second is based on using simulated visual field defects to train a deep recurrent neural network (RNN). These two methods have complementary qualities: the TFCE is neurophysiologically plausible and its output significantly correlates with Standard Automated Perimetry performed with the Humphrey Field Analyzer, while the RNN accuracy significantly outperformed the TFCE in reconstructing the simulated scotomas but did not translate as well to the clinical data from glaucoma patients. While both of these methods require further optimization, they show the potential for a more patient-friendly alternative to Standard Automated Perimetry.

## Summary

Perimetry, the mapping of the sensitivity of different visual field locations, is an essential procedure in ophthalmology. Unfortunately, Standard Automated Perimetry suffers from some practical issues: it can be tedious, requires manual feedback, and a high level of patient compliance. These factors limit the effectiveness of perimetry in some clinical populations. In an attempt to remove some of these limitations, alternatives to Standard Automated Perimetry have been tried based on tracking eye movements. These new approaches have attempted to mimic Standard Automated Perimetry, thus presenting stimuli on a fixed grid, and replacing manual with ocular responses. While this solves some issues of Standard Automated Perimetry, these approaches hardly exploit the high spatial and temporal resolution facilitated by eye-tracking. In this study, we present two novel computational methods that do tap into this potential: (1) an analytic method based on the spatio-temporal integration of positional deviations utilizing Threshold Free Cluster Enhancement and (2) a method based on training a recurrent deep artificial neural network. Our methods, based on continuous gaze tracking, provide a patient-friendly alternative to Standard Automated Perimetry and deepen our understanding of the relationship between oculomotor control and retinal sensitivity.

## Introduction

The assessment of the quality of the visual field (also called *perimetry*) is a staple of ophthalmologic evaluation. The presence of a scotoma, a region of the visual field with reduced sensitivity, is a very characteristic symptom of diseases and disorders such as macular degeneration (Tolentino et al., 1994), glaucoma (Heijl and Bengtsson, 1996), hemianopia (Williams, 1997) and several forms of retinopathy (Greite et al., 1981; Alexander et al., 2004; Voipio and Karjalainen, 2009).

The current gold standard in the diagnostic assessment of the visual field is standard automated perimetry (SAP) (Barton and Benatar, 2003). The main advantages of SAP are a thorough evaluation of multiple visual field locations, relatively easy-to-interpret results that are normalized with respect to an age-matched population, and its extensive validation in countless clinical trials and other studies. However, the approach also has several limitations: the task is complicated for people with limited cognitive capabilities, demands patient compliance (Szatmáry, 2002), and requires maintaining a stable fixation for prolonged periods of time. Furthermore, patient performance is affected by learning (Schultz, 1990; Wild et al., 2006) and fatigue (Johnson et al., 1988), as well as the expertise of the operator (Montolio et al., 2012). Together, these constraints limit the effectiveness of SAP, particularly in clinical and rehabilitation contexts such as when dealing with children (Walters et al., 2012), the elderly, and/or cognitively impaired patients (Diniz-Filho et al., 2017; Gangeddula et al., 2017).

To overcome some of these issues, various groups have implemented variants of SAP in which eye-tracking substituted the manual responses required on each trial of SAP. The most common of these approaches consists of using the saccadic reaction time to stimuli changing position as a proxy for visual sensitivity (Kim et al., 1995; Murray et al., 2009; Pel et al., 2013; Jones et al., 2019; Martínez-González et al., 2020). While this already simplifies the task, the resulting procedure still retains the trial- and grid-based approach of SAP. Therefore, the full potential of the high spatial and temporal resolution facilitated by eye tracking is not exploited.

Other approaches rely on measuring the pupillary reflex: in this method, visual sensitivity is measured as a function of the change in pupil diameter in response to flickering stimuli presented at different visual field locations (Kardon, 1992; Maddess et al., 2009; Chibel et al., 2016). This method–although more objective than SAP–is still prone to issues related to patient compliance and/or their ability to stably maintain fixation.

For these reasons, we recently proposed a novel eye-movement-based approach, inspired by the Eye Movement Cross-correlogram method and its application to measure visuospatial sensitivity (Mulligan et al., 2013; Bonnen et al., 2015). Our new method completely removes the trial-based aspect and fixation requirements of SAP in favor of a continuous assessment of oculomotor behavior over time (Grillini et al., 2018).

In our approach, the participant continuously tracks with their gaze a stimulus moving along a pseudo-random walk trajectory. The simplicity and intuitiveness of this task make it significantly more practical than other types of perimetry, irrespective of whether they require a manual or eye-movement response from the patients (Demaria et al., 2020). Furthermore, our approach provides a thorough quantification of both the spatio-temporal and statistical properties of both smooth pursuit and saccadic eye movements (Grillini et al., 2020), thus having potential applications in neurology and neuro-ophthalmology as well.

In one of our previous studies (Grillini et al., 2018), we showed that it is possible to classify a visual field defect (VFD), exclusively on the basis of the spatio-temporal properties of the eye-movements made during a short continuous tracking task. A limitation of our initial approach was that it could only classify a scotoma as belonging to one of the scotoma shape classes on which the machine classifier had been trained, and thus not reconstruct its actual location and shape. The absence of this type of information hinders a more general application of this technique in clinical and rehabilitation practice.

To overcome this limitation, here we propose two methods of analyzing continuous gaze data acquired during a tracking task that enables reconstructing the visual field including any VFD present.

Our first method is based on the intuition that, compared to a healthy participant, a patient with a VFD will make larger and more prolonged tracking errors (expressed as the distance between the positions of the eyes and the target) if the stimulus falls within their scotomatous region. In essence, the method applies threshold-free cluster enhancement (TFCE) (Smith and Nichols, 2009) to perform a spatio-temporal integration of a time series of eye-stimulus positional deviations (the signal). This results in a weighted integration of the *height* and *extent* of the signal, which in our case represent *space* (i.e., the positional deviation) and *time* (i.e., the duration of the deviation until it is corrected), respectively. Next, we reconstruct the visual field and presence of any scotoma, by back-projecting the TFCE values into visual field space. We will refer to it as the TFCE method.

The second method is based on training a recurrent deep artificial neural network. It constitutes a data-driven approach that learns features from the time-series of gaze location data collected during the tracking task. This method makes no explicit hypotheses about the underlying relationship between eye movements and scotoma characteristics and learns how the presence of a scotoma influences a participant’s visual behavior during the tracking task. The algorithm is a seven-layer recurrent neural network (RNN) whose weights are optimized to minimize its time-point-wise predictions on a set of labeled training data (obtained on the basis of gaze-contingent simulations of scotomas in known locations). Once trained, the model can accurately predict whether the distance between the eye and stimulus positions, at a given time point, is caused by the presence of a scotoma. We reconstruct the visual field and presence of any scotoma by back-projecting the RNN predictions into visual field space. We will refer to it as the RNN method.

Based on a set of simulated gaze-contingent scotoma data (Grillini et al., 2018), we show that both methods can reconstruct the shape of the VFDs. While further improvements are desirable, the methods we present here constitute a crucial stepping-stone toward the realization of truly easy and effective eye-movement-based perimetry. Our technique, which we consider to have many advantages, can complement SAP in both clinical and rehabilitation practices. Moreover, since our approach incorporates both perimetry and oculomotor evaluation in a single test, it will be of potential relevance to ophthalmologists and neurologists alike.

## Materials and Methods

**FIGURE 1:**
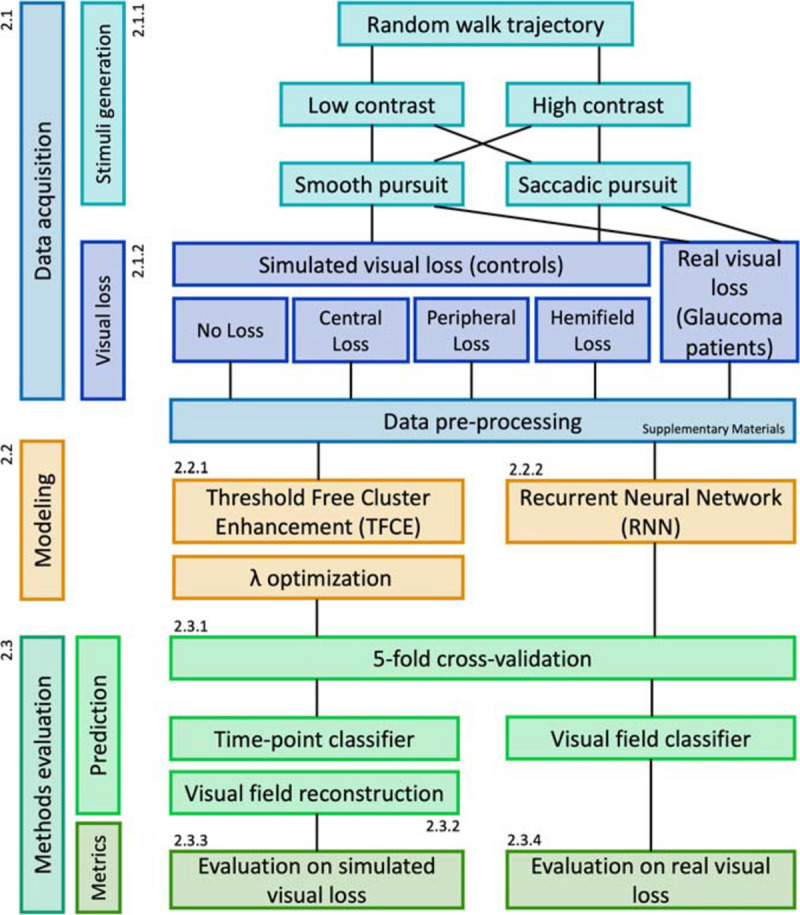
Flowchart of the experimental design.

The whole experimental procedure is illustrated schematically in Figure 1.

### Participants

We tested 50 healthy adult participants, three patients diagnosed with Primary Open Angle Glaucoma (POAG) with a visual field loss previously measured with a Humphrey Field Analyzer (Zeiss) and two additional healthy controls, age-matched with the patients. All had normal or corrected-to-normal visual acuity, verified before data collection with “FrACT” (Bach, 2007). The study followed the tenets of the Declaration of Helsinki. The Medical Ethical Committee of the University Medical Center Groningen and the Ethics Committee of Psychology of the University of Groningen approved this study. All participants provided written informed consent before participation.

### Apparatus

The experiment was designed and conducted with custom-made scripts in MATLAB using Psychtoolbox (Brainard, 1997) and Eyelink Toolbox (Cornelissen et al., 2002). The data were acquired with an Eyelink 1,000 eye-tracker (SR-Research, Kanata, ON, Canada) with a sampling frequency of 1 kHz, downsampled to 240 Hz to match the refresh rate of the stimulus display monitor Zowie xl2540 (BenQ, Taipei, Taiwan). Before each experimental session, the eye-tracker was calibrated using the built-in nine-point calibration procedures. The calibration was repeated until the average error was below 1.5°. Additional details regarding the accuracy, precision, and data loss rate of the eye-tracking measurements are presented in Supplementary Figures 1, 2.

### Data Acquisition

#### Stimuli and Conditions

**FIGURE 2:**
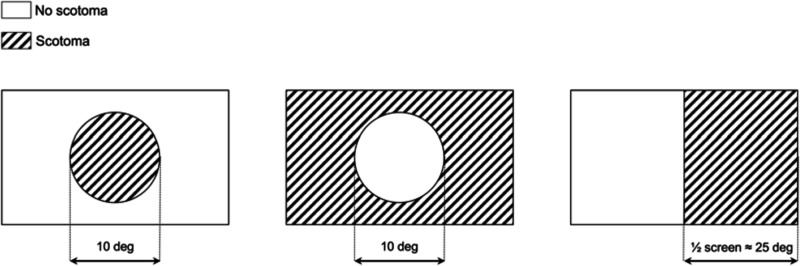
Schematic representation of the simulated visual field defects (VFD) used in this study. From left to right: central loss (scotoma size: 10°), peripheral loss (scotoma size: whole screen except for a 10° hole), hemifield loss (right half of the screen). During the experiment, the VFD was applied in a gaze-contingent manner: the center of the VFD is matched in real-time with the point of gaze of the participant, with a latency below 4 ms. This latency roughly corresponds to the inter-frame interval and ensures proper gaze-contingency.

The stimulus comprised a white dot with a diameter of 0.5° of visual angle, displayed at one of two possible contrast levels (5 and 50% from the gray background), moving along a random walk path with or without random displacements to induce saccades (the *smooth pursuit* and *saccadic pursuit* conditions, respectively). Additional detail regarding the random walk paths is available in Supplementary Materials. The point of gaze of the participants was recorded while they tracked the stimulus with their eyes. During the experiment, the 50 healthy participants of the training set were additionally subjected to different kinds of simulated gaze-contingent VFD s [no loss, central loss, peripheral loss, and hemifield loss (see Figure 2)]. Each trial, lasting 20 s, was repeated six times for each condition (2 contrast levels × 2 pursuit modalities) for a total of 24 trials and a total test time of 480 s. For the visual field reconstruction analysis, all trials are pooled together.

#### Gaze-Contingent Simulated Visual Field Loss

The simulations were obtained by superimposing in real-time (240 Hz, 4 ms delay) a uniform gray area to the current position of the participant’s gaze. The shape and size of the simulated VFD were modeled after the typical scotoma resulting from three common ophthalmologic disorders: age-related macular degeneration (central loss), late-stage glaucoma (peripheral loss), and hemianopia (hemifield loss) (Figure 2). A schematic representation of the three types of simulated VFD used in this study is shown in Figure 2.

### Modeling

#### Method #1: Spatio-Temporal Integration of Positional Deviations by Means of Threshold Free Cluster Enhancement

To simultaneously factor in the magnitude of the spatial error and its duration, we applied to our data the Threshold Free Cluster Enhancement (TFCE) (Smith and Nichols, 2009). This algorithm, originally developed for the analysis of the hemodynamic response in functional neuroimaging, specifically helps to avoid the introduction of arbitrary thresholds when performing multiple-comparison corrections. In our context, we applied the algorithm to the time-series of positional deviations *D*, where each value is the Euclidean distance between the gaze location and the stimulus position at any given time point *t*. The positional deviations as a function of time are defined in Eq. 1:

(1) $$D\left(t\right)=\sqrt{\left({\left(p_{x}\left(t\right)-s_{x}\left(t\right)\right)}^{2}+{\left(p_{y}\left(t\right)-s_{y}\left(t\right)\right)}^{2}\right)}$$

where *p*(*t*) and *s*(*t*) are the positions on the screen of the eye and the stimulus, respectively, divided into their horizontal (*x*) and vertical (*y*) components.

*D*(*t*) constitutes the input for the spatio-temporal integration performed with the TFCE equation, described in Eq. 2:

(2) $$D_{\text{TFCE}}\left(t\right)=\int_{h=h_{0}}^{h_{t}}e{\left(h\right)}^{E}h^{H}\text{d}h$$

**FIGURE 3:**
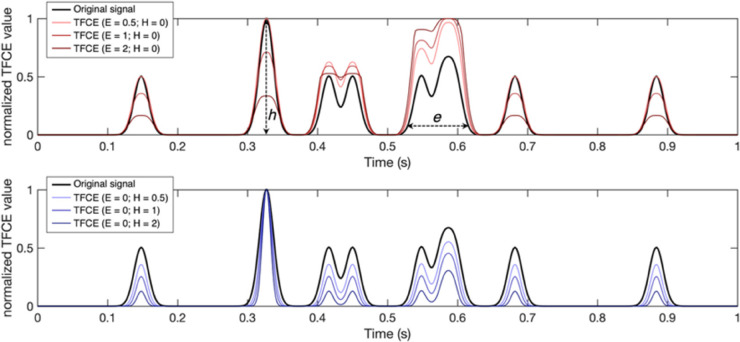
Examples of time series filtered using the threshold free cluster enhancement (TFCE) algorithm with different combinations of parameters. A larger *E* weighs more the components of the original signal with a longer temporal extent (*e*), defined as the time interval where the signal is consistently above a predefined minimum (h0). Larger *H*, conversely, weighs more the highest components of the signal in terms of intensity (*h*). Note that the TFCE values here are normalized between 0 and 1 for visualization purposes only.

Where *e* is the extent (temporal duration) and *h* is the height (spatial magnitude) of *D*(*t*) at a given point in time *t* (see Figure 3 for examples). This integral is implemented as a discrete sum using a finite step-size d*h*, [in our implementation d*h* = 1/2,500th of the maximum of *D*(*t*)]; *h*_0_ is the minimum of *D*(*t*) (which is always greater than or equal to 0), and *E* and *H* are the exponents. The resulting *D*_TFCE_(*t*) is a time-series of positional deviations weighted for their spatio-temporal integrated characteristics. Figure 3 shows some examples of using different *E* and *H* pairs: higher *E* values (red signals) enhance clusters with longer duration and suppress shorter ones; higher *H* values (blue signals) enhance the clusters with higher peaks and suppress the lower ones (Figure 3). We set these parameters to the recommended values of *E* = 2 and *H* = 0.5 (Smith and Nichols, 2009).

To label each value of *D*_TFCE_(*t*) as “healthy” or “visual loss” we apply the following algorithm.

First, to obtain the frequency distribution of all possible normative values [i.e., values of *D*_TFCE_(*t*) that should be considered healthy], *D*_TFCE_(*t*) is initially computed for every participant of the training dataset in the condition without simulated visual field loss, and the resulting values are aggregated with a histogram *F*. Next, to choose the optimal boundary to separate “healthy” and “visual loss” values, we set a threshold λ*_*n*_*, such that *F*(λ*_*n*_*) = *n*th percentile of *F*. For each value of λ*_*n*_*, we compute *B*(*t;*λ*_*n*_*) that is the binarized form of *D*_TFCE_(*t*) such that

*B*(*t*; λ_*n*_) = 0, *if D*_TFCE_(*t*) ≤ *F*(λ_*n*_) if “healthy” and *B*(*t*; λ_*n*_) = 1, *if D*_TFCE_(*t*) > *F*(λ_*n*_) if “visual loss”.

To choose the optimal λ*_*n*_* parameter for the TFCE method, we compute a 2D Spearman rank correlation between the reconstructed visual field maps and their respective ground-truth maps obtained with the known locations of the simulated scotomas. The ground-truth maps are obtained as described in section “Visual Field Map Reconstruction”, using as an input the binarized time-series using the known location of the visual field.

First, we measure the correlation between ground-truth and TFCE maps of the training set reconstructed using all possible values of λ_*n*_ = {1, 2, 3, …, 100} (i.e., one for each percentile of the histogram *F*). Then the average between participants is computed for each simulated visual loss condition, followed by the grand average across conditions. The peak of the grand average corresponds to the optimal value of λ*_*n*_* that is used to reconstruct the maps of the test data. This procedure is repeated for each fold of the fivefold cross-validation (see section “Fivefold Cross-Validation”).

#### Method #2: Recurrent Neural Networks

In this method, we train a recurrent neural network (RNN), as it is the most suitable known architecture to account for the temporal properties of the data (Rumelhart et al., 1986).

As training input **X**, we use the time series of the eye gaze positions *p*(*t*) and the stimulus positions *s*(*t*), as well as the luminance contrast (low contrast = 0; high contrast = 1) and type of pursuit of the stimulus (*smooth pursuit* = 0; *saccadic pursuit* = 1). As training output **Y**, we use both the shape of the VFD (classified as *no loss*, *central loss*, *peripheral loss*, and *hemifield loss*) of the participants that generated each training sequence and, for each time point, whether the stimulus position lies in a location obstructed by the simulated scotoma.

**FIGURE 4:**
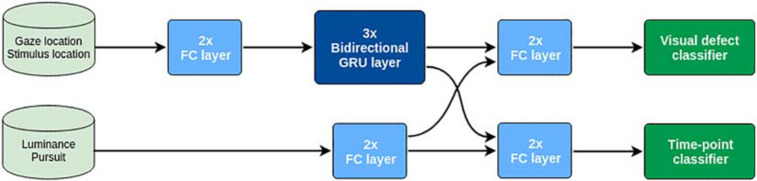
Schematic representation of the architecture of the deep recurrent neural network.

As shown in Figure 4, the network consists of two streams that initially process the sequential data [*p*(*t*) and *s*(*t*)] and categorical data (high/low stimulus luminance contrast and smooth/saccadic pursuit) separately. In particular, the sequential stream contains three bidirectional recurrent GRU layers (Cho et al., 2014) to effectively process the temporal dependencies of the sequential data. The outputs of both streams are then concatenated and used to jointly train two different softmax classifiers. One is trained to classify the shape of the VFD (*no loss, central loss, peripheral loss*, or *hemifield loss*), while the second one was trained to classify the visual field in a point-wise manner (i.e., “does the stimulus position in visual field space coordinates overlap with the scotoma?”).

We use the cross-entropy loss to define the cost function of the model, defined in Eq. 3:

(3) $$J\left(\theta \right)=-\alpha \sum_{c=1}^{M_{s}}y_{s,c}\log\left(p_{s,c}\right)-\beta \sum_{c=1}^{M_{d}}y_{d,c}\log\left(p_{d,c}\right)$$

Where *M* is the number of classes, *y* is the ground-truth label, and *p* is the predicted probability distribution, i.e., the output of each softmax classifier. Subscript *s* refers to the point-wise scotoma classifier and subscript *d* to the VFD shape classifier. In order to give priority to optimizing the reconstruction of the visual field, we set α = 0.75 and β = 0.25. The parameters θ of the model are learned through mini-batch gradient descent, using RMSprop (Tieleman and Hinton, 2012), for 15,000 iterations with a batch size of *Bs* = 128.

The training batches are formed by first selecting *Bs* different sequences from the set of 20-s trials, originally sampled at 240 Hz. Then, we randomly sampled one sub-sequence of 4.17 s (1,000 time steps) from each sequence and finally down-sample them at 60 Hz (250 time steps). The stimulus contrast level and pursuit modality of the corresponding sequences are also added to the training batches.

This deep model can be regarded as a mapping *y* = *f*(*x*;θ), where *y* = [pw*_*s*_* pw*_*d*_*], pw*_*s*_* being the point-wise scotoma prediction and pw*_*d*_* the VFD shape prediction of a sub-sequence *x*. In order to classify the VFD shape of one participant, we are interested here in pw*_*d*_*.

Since the data acquisition for one participant consists of six repetitions of 20 s trials for each contrast/pursuit combination, we average the predicted output probability distributions of multiple sub-sequences. In particular, we average the predictions of the *K* = 6 × 2 × 2 = 24 downsampled sequences. The predicted VFD for a participant *s* is thus defined by Eq. 4:

(4) $$\hat{y}=\underset{c}{\arg\max}\frac{1}{K}\sum_{i:x_{i}\in S}f\left(x_{i};\theta \right)$$

Where *K* is the number of subsequences in the set of trials *S* of participant *s*.

### Methods Evaluation

#### Fivefold Cross-Validation

To assess the quality of visual field reconstruction using the TFCE- and RNN-based methods, we carried out a participant-aware fivefold cross-validation. To do this, we split the data from the 50 participants into five sets, each containing the data from 40 participants for training and 10 participants for testing. We ensured that, in each fold, the sets of participants for training and testing are always disjoint. An example of data partitioning is as follows:

• Fold 1:
  • Train: participants [1, 2,…, 40]
  • Test: participants [41, 42,…, 50]
• Fold 2:
  • Train: participants [1, 2,…, 30] U [41, 42,…, 50]
  • Test: participants [31, 32,…, 40]
• Fold 3:
  • Train: participants [1, 2,…, 20] U [31, 32,…, 50]
  • Test: participants [21, 22,…, 30]
• Fold 4:
  • Train: participants [1, 2,…, 10] U [21, 22,…, 50]
  • Test: participants [11, 12,…, 20]
• Fold 5:
  • Train: participants [11, 12,…, 50]
  • Test: participants [1, 2,…, 10].

To evaluate the feasibility of our methods in a clinical setting, we additionally assessed three participants diagnosed with primary open-angle glaucoma (POAG) and two age-matched healthy control participants. These participants were not part of any training set.

#### Visual Field Map Reconstruction

To reconstruct the visual field maps, the classified time-series need to be converted into visual field coordinates. Both the TFCE and the RNN outputs consist of binarized time-series *B*(*t*) where each entry has a value of 0 if it is classified as not being obstructed by a scotoma and 1 if it is. Each entry also has associated with it a pair of *xy* coordinates, where *x* is the difference between the horizontal gaze and stimulus positions at that time point and, analogously, *y* is the difference between the vertical gaze and stimulus positions. These are retinotopic coordinates, meaning that they represent where the stimulus was with respect to the gaze of the participant. These coordinates are then binned into an N × M grid, where each square represents 1°^2^ of visual space and N and M are the dimensions of the visual field tested. Each square contains the percentage of occurrences that that specific location has been classified as being obstructed by a scotoma and gets color-coded accordingly. For visualization purposes, these retinotopic coordinates can be easily converted into polar coordinates.

**FIGURE 5:**
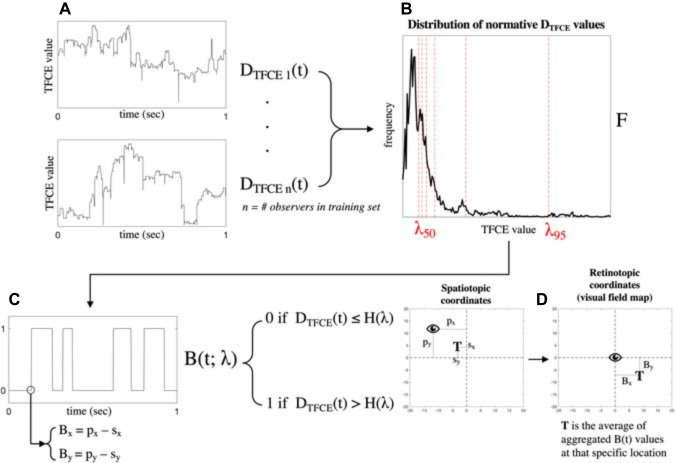
Schematic representation of the algorithm pipeline. Starting from the TFCE-filtered time-series in panel **(A)**, a probability distribution *F* of all possible normative TFCE values is computed in panel **(B)**. For all percentiles λ*_*n*_* of the distribution *F*, a threshold value *F*(λ*_*n*_*) is defined and used to binarize the TFCE-filtered signal in panel **(C)**. Each time point of the TFCE-filtered signal has associated with it the spatiotopic coordinates of gaze (*p*_*x*_ and *p*_*y*_) and the stimulus positions (*s*_*x*_ and *s*_*y*_), which are converted into retinotopic coordinates *B*_*x*_ and *B*_*y*_. The resulting mapped retinotopic coordinates in panel **(D)** are associated with a target location *T* which contains the average of the aggregated *B*(*t*) values at that specific location. *B*(*t*) is the expected probability that that specific location is affected by a scotoma. An analogous back-projection algorithm is implemented for the reconstruction of the visual field using the recurrent neural network (RNN), where the binarized time-series in panel **(C)** is defined by the output of the model instead of the threshold *F*(λ*_*n*_*).

A summary of the pipeline for visual field map reconstruction from gaze-tracking TFCE-filtered time-series is shown in Figure 5. The RNN visual field map reconstruction is analogous, with the binarized values of *B*(*t*) being provided by the outcome of the *time-point classifier* (Figure 4) rather than the threshold value *F*(λ_*n*_)applied to TFCE-filtered eye-tracking signals.

Note that the back-projection into visual field space has been used to reconstruct the *ground-truth* maps as well. In their case, the information about the presence or absence of the scotoma is known a priori, but the reconstruction is still necessary to ensure a proper spatial comparison between the *ground-truth* and the *TFCE* or *RNN* maps since the tracking behavior is always different due to the random nature of the paths.

#### Evaluation on Simulated Visual Field Defects

After reconstructing the visual field maps, we evaluated the performance of the TFCE method and RNN “time-point classifier” by computing a 2D Spearman rank correlation between the ground-truth maps and the reconstructed maps. This analysis is done for each fold independently, so that any subject belonging to the test set is excluded from the training set.

Furthermore, we used the “visual field classifier” of the RNN to assess the robustness of categorical classification of VFD s in case of heavy miscalibrations of the eye-tracking setup (see Supplementary Materials, section “Testing Robustness to Miscalibration Errors”).

#### Evaluation on Patients Data

To provide a proof-of-concept of the viability of these methods in a clinical setting, we asked three patients and two healthy control participants with various degrees of visual field loss to perform the visual tracking task. We then compared the maps obtained with TFCE and RNN to those obtained with SAP using the Humphrey Field Analyzer (HFA), using the SITA-Standard algorithm. The HFA was performed monocularly on the eye affected by POAG in the case of patients and on the dominant eye in the case of controls. We then compared the mean deviation (MD) as reported by the HFA (MD_HFA_) with an MD computed based on the TFCE- and RNN-reconstructed maps (MD_TFCE_ and MD_RNN_, respectively).

Since the stimulus used for the tracking task followed a random-walk path, we could not ensure complete coverage of the whole visual field. Therefore, the computation of the MD_TFCE_ and MD_RNN_ values comprises a correction for visual field coverage. Our MDs are computed as follows:

(5) $$\text{MD}=\left[\frac{1}{n^{\prime }}\cdot \sum_{i=1}^{n^{\prime }}T\left(B_{x},B_{y}\right)\right]\cdot \frac{n^{\prime }}{n}$$

Simplified as:

(6) $$\text{MD}=\frac{\sum_{i=1}^{n^{\prime }}T\left(B_{x},B_{y}\right)}{n}$$

Where *n’* is the number of visual field locations sampled, *n* is the number of all possible visual field locations and *T*(*B_*x*_, B_*y*_*) is the probability that a specific location is affected by visual loss (see Figure 5).

## Results

**FIGURE 6:**
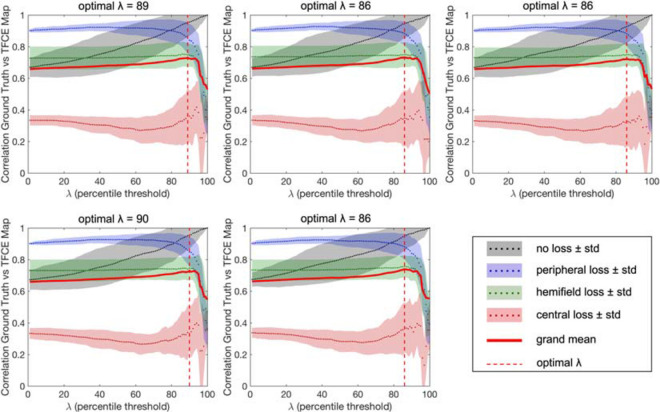
Results of the optimization of the λ parameter for each fold of the cross-validation. The optimal percentiles to be used as thresholds between “healthy” and “impaired” TFCE values are in the range 86th–90th (mean 87.4), corresponding to the peaks of the grand average between all tested conditions.

**FIGURE 7:**
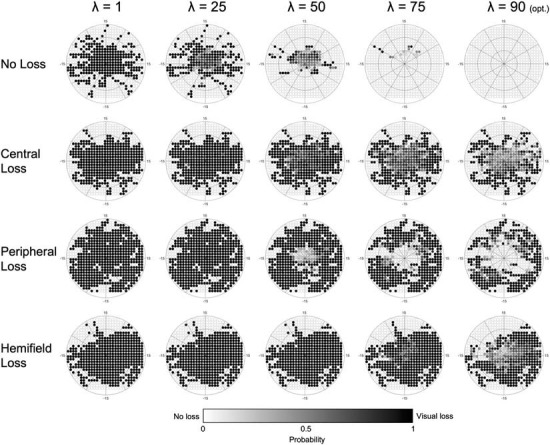
Example of the effect that adjusting the λ parameter has on a single participant, across all conditions. As the optimal λ (90) is approached, the number of false positives across all conditions is minimized. Darker colors indicate a higher probability of that point being affected by visual loss (white = 0%, black = 100%).

The results of the optimization of the parameter λ*_*n*_* based on the maximum average accuracy of each condition for each fold are shown in Figure 6, while Figure 7 shows the effect that adjusting λ*_*n*_* has on the visual field map reconstruction of a participant across all conditions.

The results are very consistent across the fivefolds, with only the central loss condition showing minimal variability. The resulting optimal λ*_*n*_* is the average threshold determined for each of the five folds (#1: 89; #2: 86; #3: 86; #4: 90; and #5: 86).

**FIGURE 8:**
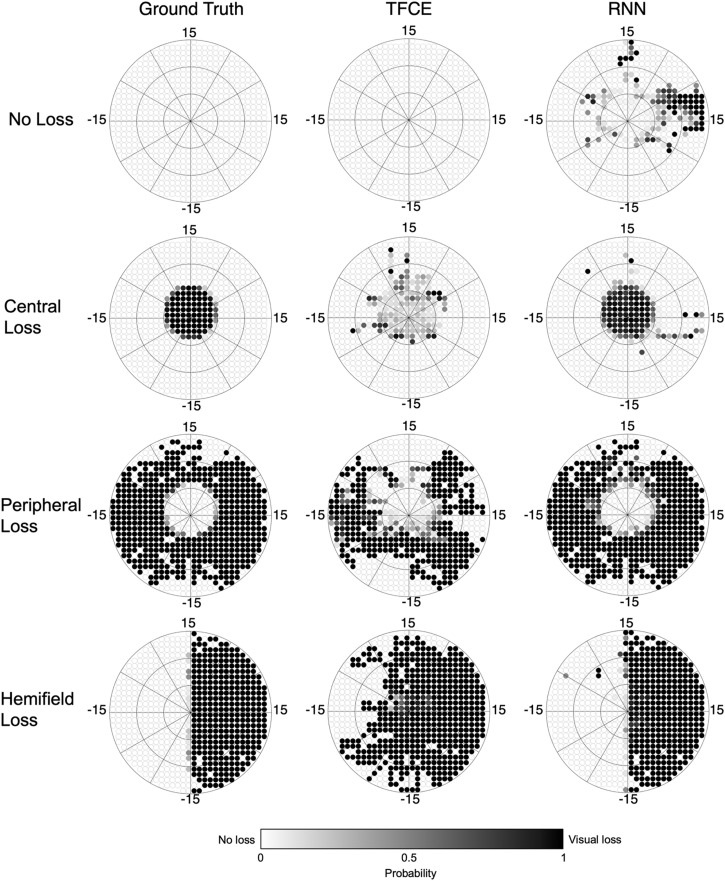
Reconstructed visual field maps for a single participant. Darker color indicates the higher probability of that point being affected by visual loss (white = 0%, black = 100%). The TFCE maps, although less accurate than the RNN ones, still allow the recognition of the shape of the underlying scotomas. The RNN maps, on the other hand, closely resemble the ground-truth maps.

Next, in the test set, we reconstruct maps using both the TFCE (with the optimized λ*_*n*_*) and RNN methods. For each method separately, we compute the 2D Spearman rank correlation with the respective ground-truth reconstructed map. Figure 8 shows examples of reconstructed visual field maps for all simulated visual loss conditions applied to one random participant of the test set.

We evaluated the overall performance of both the TFCE and RNN methods for all conditions tested, applying the fivefold cross-validation for both methods using the same 40–10 split. Each fold uses its own optimized λ*_*n*_* as shown in Figure 6.

**FIGURE 9:**
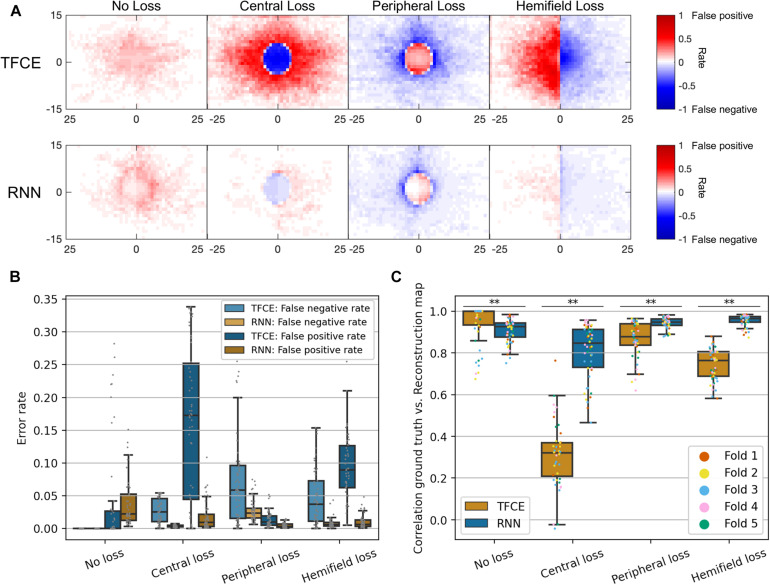
**(A)** Spatial distributions of false positives and false negatives rates for each condition and for both TFCE and RNN methods. **(B)** Total error rates combining false positive rate and false negative rate of TFCE and RNN. **(C)** Comparison between TFCE and RNN visual field map reconstruction accuracies. The RNN method shows higher correlations with the ground-truth as well as being more consistent than the TFCE method. Statistically significant differences (*p* < 0.001) are found across all tested conditions. Error bars show the 10th and 90th percentiles of each distribution.

Figure 9 shows the performance for both the TFCE and RNN methods, quantified with the spatial distribution of False Positives and False Negatives (Figure 9A), the False Positive Rate/False Negative Rate (Figure 9B), and with their accuracy, computed as the 2D Spearman rank correlation between the reconstructed and ground-truth maps (Figure 9C).

A Kruskal-Wallis test (nonparametric one-way ANOVA) shows a statistically significant difference between the two methods for all observed conditions (all *p*-values < 0.001), where the RNN method proved to be more accurate than the TFCE in all but one condition (*No Loss*). Overall, the RNN method also showed less variability between participants (see Table 1).

**TABLE 1:** Variability of the two methods threshold free cluster enhancement (TFCE) and recurrent neural network (RNN) measured with interquartile ranges (IQR) of the accuracies of the visual field map reconstructions.

	No	Central	Peripheral	Hemifield	Average
	**loss**	**loss**	**loss**	**loss**	
Accuracy TFCE	0.9455	0.3122	0.8664	0.7435	0.7169
Accuracy RNN	0.9070	0.8066	0.9429	0.9555	0.9030
IQR TFCE	0.0693	0.1651	0.1038	0.1138	0.1141
IQR RNN	0.0693	0.1863	0.0303	0.0268	0.0782

Finally, to evaluate the generalizability of our approaches to a real clinical context, we compared the TFCE and RNN methods with the perimetry maps obtained with the Standard Automated Perimeter HFA, the current gold standard in perimetry. For this comparison, we tested participants with real VFD s of different severity and compared the MD_HFA_ to the MD_TFCE_ and MD_RNN_ computed as described in paragraph *Application of eye-tracking visual field reconstruction to clinical data* in section “Materials and Methods”. For this comparison, we took as optimal TFCE λ*_*n*_* the average between the optimal values of each fold. This λ*_*n*_* corresponds to the 87.4th percentile of the distribution of all possible TFCE normative values. The clinical participants have been treated as a new independent test set, no re-training of the RNN model has been performed.

Contrary to the simulated scotomas case, the TFCE method outperformed the RNN in terms of agreement with the MD values of the HFA, showing a significant rank correlation between MD_TFCE_ and MD_HFA_ ($R_{\text{TFCE}}^{2}=0.88$, *p*_TFCE_ = 0.0117;$R_{\text{RNN}}^{2}=0.49$, *p*_RNN_ = 0.112).

## Discussion

Our main conclusion is that it is possible to reconstruct visual field maps, including the location of a scotoma, based on eye-tracking data acquired with a method of continuous gaze tracking. We consider this a breakthrough proof-of-principle, as it indicates a pathway toward the design of a high-resolution, patient-friendly way to perform perimetry. Below, we discuss the merits (and limitations) of the two methods for visual field map reconstruction that we presented and tested in this study: (1) spatio-temporal integration of positional deviations performed with TFCE and (2) recurrent deep artificial neural network (RNN). Moreover, we will compare our techniques to other proposed methods for eye-tracking-based perimetry and discuss possible further improvements.

### Continuous Gaze-Tracking Allows the Reconstruction of Visual Field Maps

#### Threshold Free Cluster Enhancement

The spatio-temporal integration of positional deviations via TFCE allows the reconstruction of visual field maps without requiring any prior knowledge about VFDs. It is a method easy to implement, computationally inexpensive, and biologically-plausible: a loss of sensitivity in the visual field is associated with lower accuracy and higher delays of the eye-movements landing in the impaired region. This is consistent with findings from previous studies involving patients with central (Van der Stigchel et al., 2013) and peripheral (Burton et al., 2014) VFDs.

The TFCE method performed quite well in reconstructing visual field maps with no loss or with peripheral loss (accuracies of 0.95 and 0.87, respectively), while it fared less optimally in reconstructing central losses and hemifield losses (accuracies of 0.31 and 0.74, respectively). A plausible reason for this discrepancy is the heavy foveal bias that is inherent to continuous tracking tasks: to accurately track the stimulus the observer must keep it as close as possible to the centermost part of their visual field. In the case of occlusion of the fovea, this is not possible, leading to prolonged errors that never allow the positional deviations to return to zero (i.e., when the eye is on the target). In the TFCE algorithm, this results in an erroneous definition of the baseline (the *h*_0_ parameter in Eq. 2) which in turn leads to an increased error rate toward the center of the visual field for *central loss* and *hemifield loss* conditions (Figures 9A,B).

Furthermore, although this method fares well in detecting a scotoma in its expected location, it struggles in precisely defining the edges of the defect (for examples see Figure 8, second and fourth rows). This can be due to the presence of compensatory eye movement strategies in the observer or the use of a different preferred retinal locus in the presence of VFDs, whether real (Coeckelbergh et al., 2002) or artificial (Cornelissen et al., 2005; McIlreavy et al., 2012).

**FIGURE 10:**
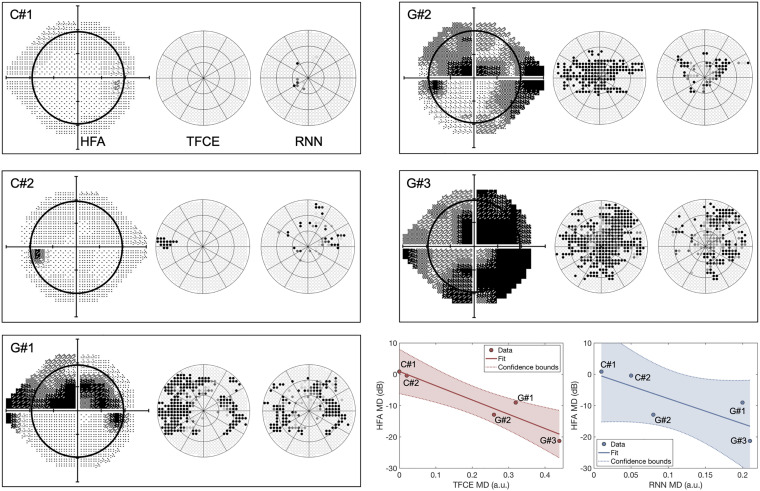
Perimetric maps of the five patients tested with our continuous tracking test. The TFCE maps are reconstructed using as λ*_*n*_* the average of the values obtained by each fold (see Figure 6). C#1 and C#2 are healthy controls, while G#1, G#2, and G#3 and patients previously diagnosed with Primary Open Angle Glaucoma. The black circle within the HFA maps represents the portion of the visual field covered by the TFCE and RNN maps. The TFCE method shows a significant correlation between its Mean Deviation index and the one obtained with the HFA, whereas the RNN method does not ($R_{\text{TFCE}}^{2}=0.88$, *p*_TFCE_ = 0.0117; $R_{\text{RNN}}^{2}=0.49$, *p*_RNN_ = 0.112).

Despite these limitations, the maps reconstructed with this method corresponded well to those reconstructed using SAP (Figure 10), and their respective MD values significantly correlated with each other, showing promising potential for generalizability into clinical use.

#### Recurrent Neural Network

The reconstruction of visual field maps employing the RNN method proved to be highly accurate with simulated scotomas, with an average accuracy across conditions above 0.90. This method, however, did not show significant correlations with the maps of POAG patients obtained using SAP. This could be due to the way the network was trained.

Using a limited number of predefined scotoma shapes made the RNN optimal at reconstructing similar scotomas (with an average accuracy above 0.90), but not as effective in dealing with new shapes not encountered before. This is also evident from the spatial distributions of false positives and false negatives (Figure 9A, lower panel), where the RNN reveals having a clear internal representation of the four scotoma shapes used in the training data.

Another aspect that limits the implementation of the RNN in a clinical setting is that, in its current form, it requires training data for which the location of the scotoma is known. This is easily achievable using simulated gaze-contingent scotomas, but with actual patients, it is impossible to establish their objective ground-truth. While the comparison to SAP is an obvious approach as it is the current gold standard, this method can be unreliable when testing moderate to severe visual loss (Gardiner et al., 2014), cognitively impaired (Kim et al., 1995; Murray et al., 2009; Pel et al., 2013; Diniz-Filho et al., 2017; Jones et al., 2019), or very young patients (<6 years old) (Tschopp et al., 1998; Patel et al., 2015), leading to a distorted ground-truth that would not constitute good training data.

We must note though that most of these limitations can potentially be overcome by redesigning the way the training data is acquired. In section “Current Limitations and Future Improvements”, we propose possible solutions to the training issue and other problems.

### Clinical Relevance of Continuous Gaze-Tracking Perimetry

The two computational methods to derive perimetric information proposed in this study showed mixed outcomes when compared to the outcome of Standardized Automated Perimetry (SAP) as done with a Humphrey Field Analyzer (HFA). While the MD derived from TFCE-reconstructed maps significantly correlated with the MD obtained from SAP, the MD derived from the RNN-reconstructed maps did not. However, the TFCE method showed overall lower accuracy compared to the RNN in the simulated visual field loss conditions. This may lead one to question the present approach. However, when comparing it to SAP, the clinical relevance of continuous gaze-tracking and its associated computational methods must be evaluated while considering a number of aspects. The first aspect to consider is the biological plausibility of the method. Our TFCE approach assumes a relationship between retinal sensitivity at a given location and the oculomotor delay toward said location. This relationship has been confirmed in patients with peripheral or central scotomas (Van der Stigchel et al., 2013; Burton et al., 2014) and it is the basis of many other proposed forms of eye-movement-based perimetry (Kim et al., 1995; Murray et al., 2009; Pel et al., 2013; Jones et al., 2019; Martínez-González et al., 2020). This implies that in its current form, the TFCE approach could already be deployed in a clinical setting (perhaps after optimization of its hyperparameters, see section “Current Limitations and Future Improvements”). In contrast, the RNN approach lacks this *a priori* biological plausibility. While this does not exclude it from being clinically applicable, it does imply that the data used to train the network must be sourced with great care to ensure biological plausibility and clinical relevance. Further optimizing the RNN will require collecting more and in particular more variegated simulation data.

The second aspect to consider is the difference between static and kinetic perimetry. The visual stimulation used in continuous gaze-tracking perimetry is more analogous to kinetic perimetry (e.g., as performed by means of a Goldman Perimeter) than to the static stimuli used in SAP. When comparing the results of static and kinetic perimetry, it is well documented that their visual field maps do not always match, a phenomenon known as Stato-Kinetic Dissociation (SKD). The basis of SKD can be either physiological (Hudson and Wild, 1992; Osako et al., 1997) or pathological (Safran and Glaser, 1980; Hayashi et al., 2017). This implies that the maps derived from continuous gaze-tracking perimetry and SAP may reflect different aspects of visual sensitivity. On the one hand, continuous gaze-based perimetry may emphasize the sensitivity of the magnocellular system, while SAP may emphasize that of the parvocellular system (Safran and Glaser, 1980). If so, this would imply that continuous gaze-tracking perimetry and SAP would best be seen as complementary approaches.

The final aspects to consider are comprehensiveness and ease of use. While SAP does only perimetry, the use of eye movements has two important clinical advantages: the data acquired for perimetry can simultaneously be used for additional neuro-ophthalmic evaluations (Grillini et al., 2020). Moreover, according to patients, continuous gaze-tracking perimetry is less tiring and easier to perform than SAP (Demaria et al., 2020). These properties could make continuous gaze-tracking perimetry an interesting approach for preliminary screenings.

### Comparison With Existing Tools for Eye-Tracking-Based Perimetry

The rationale behind the development of a perimetric tool based on continuous gaze tracking is rooted in the previous evidence that kinetic perimeters, i.e., devices such as the Goldmann Perimeter and the Octopus 900 where the probing stimulus is moving, outperform SAP both in reliability and ease of use in patients aged 5–8 (Patel et al., 2015). This suggests that the use of moving stimuli in perimetry, although considered not optimal for the general population, might be relevant within specific clinical contexts, and eye-tracking techniques constitute a fertile ground to explore this possibility.

We are not the first to propose eye tracking as a means toward removing some critical aspects of SAP, such as its high cognitive load and the need for manual feedback (Kim et al., 1995; Murray et al., 2009; Pel et al., 2013; Jones et al., 2019). In fact, even the counting of fixation losses and determining blink frequency can be seen as an elementary form of eye-tracking that is used to improve the reliability of SAP (Ishiyama et al., 2015; Asaoka et al., 2019).

So far, all existing tools for eye-tracking-based perimetry employ the same working principles of conventional perimetry with the primary difference being using ocular responses instead of manual ones. The patient is still asked to repeatedly answer the question “do you see the stimulus?”, and the answer is provided by the landing (or not) of a saccade within a Region-of-interest (ROI) around the target (Kim et al., 1995; Jones et al., 2019), or by the latency of a saccade that fell within the ROI of a displaced target (Pel et al., 2013).

This approach comes with the advantage of allowing a precise sensitivity threshold estimation for each tested visual field location but has two major downsides. First, since each point needs to be tested individually and repeatedly, the spatial resolution is intimately interrelated to the available testing time. Second, discrete eye movement perimetry is heavily reliant on optimal instrument calibration: if the average calibration error exceeds the ROI radius of each target, it is very well possible to have completely invalid maps where none of the measured locations on the visual field reflects the true underlying visual sensitivity.

Our method based on continuous tracking is designed to be less affected by these sampling- and calibration-related issues.

First, as each time sample contributes to the final map and spatial binning is applied only *a posteriori*, even without any smoothing, it is possible to obtain detailed maps (see Figures 6, 7, 9).

Second, although less affected by the calibration issues that might arise during a clinical evaluation, our gaze-based method of visual field mapping can still allow for the classification of VFDs. Our method is based on the same stimuli as used in a previous study by Grillini et al. (2018), where spatio-temporal features were used to classify the shape of the underlying VFD. The categorical classification in that study was performed by training a simple decision tree with the features explicitly extracted from the gaze and stimulus data. The temporal features are affected minimally by poor calibration and still yield sufficient information about the type of scotoma.

In the present study, these spatio-temporal features of eye movements are not made explicit, but their categorical classification is still performed by our neural network (see Figure 4, upper stream). As the temporal dependencies are taken into account by the long short-term memory properties of the bidirectional GRUs layers (Cho et al., 2014), the network can perform a satisfactory VFD classification (not reconstruction) also in the presence of rather poor calibrations. We provide empirical evidence for this claim in Supplementary Materials, where we show how the RNN is robust even in the presence of rather severe distortions applied to the data. The performance of the RNN remains above chance-level (25%) for absolute distortions in the data up to 5° (see Supplementary Figures 3, 4), with an accuracy above 60% up until 3°.

### Current Limitations and Future Improvements

While we believe that the results presented constitute a promising proof-of-concept of the viability of continuous-gaze-tracking perimetry, their implementation into a clinical setting would still require several improvements, both in the acquisition and analysis of gaze data.

First, we trained the RNN using simulated VFDs to establish a ground-truth for the presence vs. absence of a scotoma. While this is not feasible with real scotomas, an alternative is to train the RNN with more realistic and diverse visual loss simulations. The ones that we used were either masking the stimulus completely or not at all, as well as having stereotyped shapes lacking all the idiosyncrasies that can be present in actual visual field impairments. Training the RNN with different “archetypal” shapes with multiple levels of contrast reduction could provide a significant improvement over our present results (Elze et al., 2015). Another re-training possibility could use data augmentation in a similar way to what we show in Supplementary Materials: the RNN is already robust to miscalibration error, but its performance could improve further if the training data is augmented with such perturbations. Data augmentation has proven to be one of the most effective methods to improve the robustness of neural networks to noise and other data perturbations (Hernandez-Garcia et al., 2019; Rusak et al., 2020).

Second, the TFCE method requires two parameters to be defined beforehand: *H* and *E*, representing the weights to be attributed to the height and the extent of the positional deviations, respectively. These parameters are typically chosen empirically (Smith and Nichols, 2009), while a future implementation of this method can comprise a preliminary optimization phase in which the parameters *H* and *E* are chosen analytically using machine learning. Possible computational approaches for the optimization of the TFCE hyperparameters would be grid-search cross-validation (Abu-Mostafa et al., 2012) or Bayesian optimization (Brochu et al., 2010).

Third, optimization of the stimulus properties. The current stimulus is the same used to extract the spatio-temporal properties of eye movements for neuro-ophthalmic screening (Grillini et al., 2020), but it can be further optimized to perform visual field assessment. For instance, properties such as luminance contrast and speed can be adjusted to “lengthen the tail” of the normative distribution of TFCE values (see Figure 5B), thus facilitating the thresholding between “healthy” and “impaired” values. We hypothesize that adopting test conditions closer to the sensory limits may help in this (i.e., using a stimulus contrast close to the contrast-sensitivity threshold and using an average speed above 30°/s to make smooth pursuit more difficult).

Analogously, the trajectory of the moving target can be adjusted with the goal of maximizing visual field coverage and minimizing central bias. For example, the positions to which the target will “jump” can be drawn from a pattern similar to the stimulus locations of SAP, rather than being randomly chosen. This would ensure optimal coverage while retaining the advantage of a higher spatial resolution brought by continuous gaze-tracking.

Fourth, the present simulated visual loss data used for training the RNN (healthy controls) and the clinical data (glaucoma patients) used to test it were acquired from two groups with different age ranges (20–30 vs. 70–80). The different age ranges may have affected eye movements even in the absence of a clinical condition (Rottach et al., 1996).

Lastly, an additional improvement could be to take into account the physiology of the retina and model *H* and *E* according to the “hill of vision” (Jacobs and Patterson, 1985). In this case, different values of *H* and *E* could be determined for sections of the retina at different eccentricities, such as the central peak (0°–10°), the mid-plateau (15°–25°), and the peripheral decay (above 25°) and combined with the hyperparameter optimization methods mentioned above for best results.

## Conclusion

We developed and proposed two methods that enable the reconstruction of visual field maps by estimating retinal sensitivity using continuous gaze-tracking data: (1) spatio-temporal integration of positional deviations performed with TFCE and (2) recurrent deep artificial neural network (RNN). The two methods possess complementary qualities (and downsides): the TFCE is biologically-plausible and computationally efficient while the RNN is remarkably accurate when provided with proper training data. We conclude that both methods can contribute to making gaze-based perimetry more viable in the future.

## Data Availability Statement

The raw data supporting the conclusions of this article will be made available by the authors, without undue reservation.

## Ethics Statement

The studies involving human participants were reviewed and approved by Medical Ethical Committee of the University Medical Center Groningen and Ethics Committee of Psychology of the University of Groningen. The patients/participants provided their written informed consent to participate in this study.

## Author Contributions

AG and FC designed the study. AG, AH-G, and RR developed the methods and wrote the software. AG and GD collected the data. AG and AH-G analyzed the data. AG, AH-G, and FC wrote the manuscript. All authors reviewed the manuscript.

## Conflict of Interest

AG, AH-G, and RR listed as inventors on the patent application (Grillini et al., 2019). AG majority shareholder of REPERIO, BV, a private company that develops ophthalmic and neurological tests based on eye-movements. The remaining authors declare that the research was conducted in the absence of any commercial or financial relationships that could be construed as a potential conflict of interest.
