# Supplementary material for: Computational Methods for Continuous Eye-Tracking Perimetry Based on Spatio-Temporal Integration and a Deep Recurrent Neural Network
Source: Front Neurosci. 2021 Apr 29;15:650540. doi: 10.3389/fnins.2021.650540 (PMC8117233; doi:10.3389/fnins.2021.650540)
Supplement: Supplementary file 1 [file Data_Sheet_1.pdf]

Supplementary Materials for

"Computational methods for continuous eye-tracking perimetry based on spatio-temporal integration and a deep recurrent neural network"

Alessandro Grillini, Alejandro Hernández-García, Remco J. Renken, Giorgia Demaria, Frans W. Cornelissen

Details on the generation of the stimuli's random walk paths

The stimulus trajectory consists of a constrained, random path. The two constraints are: (1) the stimulus trajectory must stay within the boundaries of the screen. (2) The stimulus trajectory cannot contain periodic autocorrelations.

The stimulus trajectory is constructed by generating an array of velocity values where, at each time-point, the velocity values for the horizontal and vertical components are drawn from a Gaussian distribution. The temporal frequency of the array of velocity values is designed to match the refresh rate of the monitor where the stimulus is displayed (240 Hz in this study).

The distribution of the velocity values is always zero-meaned, and its standard deviation can be adjusted to modulate the resulting velocity of the displayed stimulus. The values used in this study are sigma = ~ 64 deg/sec for the horizontal component and ~32.33 deg/sec for the vertical component. These values have been chosen empirically, to fit the screen's aspect ratio and to

produce a stimulus sufficiently hard to follow for healthy observers while challenging, yet not impossible to follow, for visually impaired observers.

The velocity array is low-pass filtered (cut-off frequency = 10 Hz) by convolution with a Gaussian kernel such that excessive jitter is minimized. Subsequently, via temporal integration, velocities are transformed into positions of the stimulus. In order to induce the observer to also perform saccadic movements in addition to the smooth pursuit, we created trajectories with random stimulus displacements. This is achieved by randomly juxtaposing epochs of 2 seconds each taken from the original 6 smooth trajectories. The resulting saccadic distributions are shown in Figure S1.

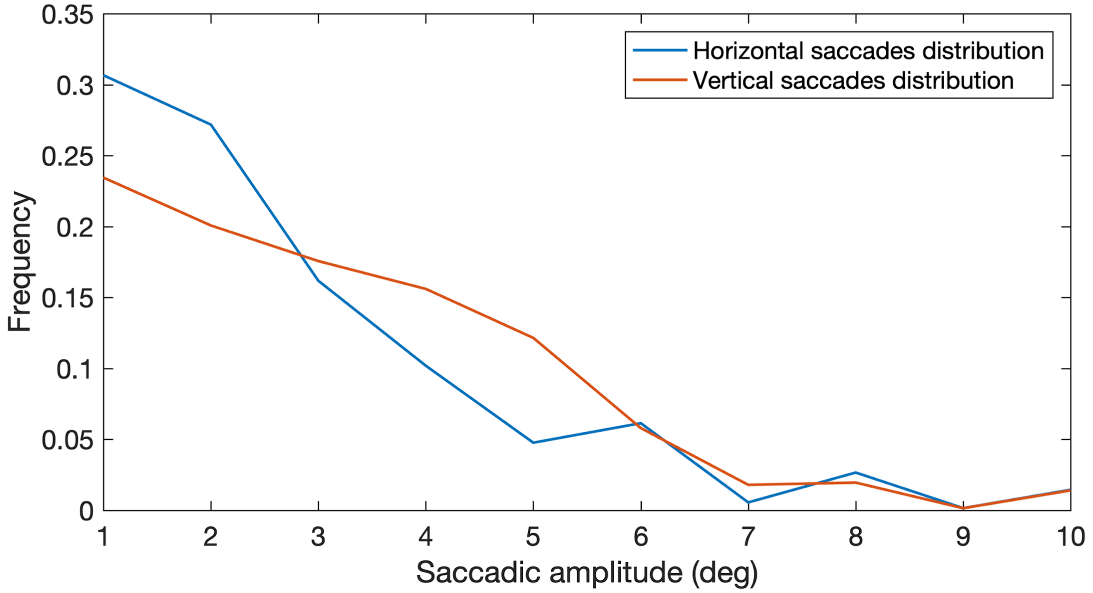

Figure S1. Distributions of saccadic “jumps” amplitudes in the stimuli’s trajectories.

During a typical assessment, each observer is presented with 6 different trajectories of 20 seconds each per pursuit modality, one being with and the other without saccadic insertion, usually referred to as *smooth* and *saccadic pursuit* conditions, respectively.

Pre-processing of eye-tracking data

The data acquired consists of time series of eye gaze positions $p(t) = \begin{bmatrix} p_x \\ p_y \end{bmatrix}$ expressed in visual field coordinates.

Blinks and other artifacts are removed as follows: blink periods are identified by spikes in the vertical gaze velocity (first derivative of p_y > 300 deg/sec) followed by a plateau (first derivative of $p_y = 0$) or missing data. This specific artifact is caused by how video-based eye-trackers compute gaze position: when the eyelid is closing due to blinking, it partially covers the pupil, which is erroneously interpreted as a rapid shifting upwards. The closed eye is then recorded as missing data or as the last valid position recorded. Each blink period found is dilated by 5 samples on both sides. If the total data loss (due to blinks or otherwise) exceeds 25% of the trial duration, the entire trial is removed from further analysis. Lastly, the data in the blink-period is imputed by fitting an autoregressive model (Akaike, 1969) using 10 samples preceding and following each of the above-defined blank periods. After all blinks are removed and missing data are filled, a Butterworth low-pass filter (half-power frequency = 0.5 Hz) applied to $p(t)$ is used to remove any instrument noise from the recorded gaze positions.

Eye-tracking data quality control

When dealing with eye-tracking recordings from different clinical populations, it is important to ensure that the outcomes of the analysis are truly dependent on the oculomotor performance alone and not on data acquisition issues that might have arisen due to collateral effects of the clinical condition in itself. In our study, we measured the eye movements of Primary Open Angle

Glaucoma patients and healthy controls (training dataset and age-matched controls) with the intent of using their oculomotor performance during a tracking task to measure their visual field.

To evaluate the robustness of our measures, we computed three parameters: accuracy, precision, and data loss rate. To benchmark our data we used the time series obtained in the *smooth pursuit* condition with *high contrast*. We chose this condition since it is the easiest condition to track, so that eventual perceptual issues will not create a confounding factor in measuring the quality of eye-tracking data.

The accuracy of the eye-tracking data is computed as described in Equations S1 and S2:

Eq. S1

$$p_c(t)[n] = p(t)[\langle n - n_0 \rangle_N];$$

Eq.S2

$$A = min\{ (\sum_{1}^{N} \frac{|s(t) - p_c(t)_{n_0=1}|}{N}, \sum_{1}^{N} \frac{|s(t) - p_c(t)_{n_0=2}|}{N}, \dots, \sum_{1}^{N} \frac{|s(t) - p_c(t)_{n_0=100}|}{N})\}$$

Where Eq.S1 defines the circularly shifted time-series $p_c(t)$ of the time-series $p(t)$, which is the vector of gaze positions. N is the length of the time-series and represents the modulo of the circular shift, while n_0 is an integer that defines the step of the shift and in our case can assume values from 1 to 100. So, the nth element of $p_c(t)$ is the n-n_0^{th} element of p(t) for every shift with step size from 1 to 100. Eq.S2 defines accuracy A the minimum of the array where each element is the average absolute error, calculated as the difference between stimulus positions s*(t)* and gaze positions $p_c(t)$, circularly shifted towards the left for a maximum of 100 steps. A circular shifting towards the left discounts the oculomotor lag present in every tracking session and ensures that the average absolute error is computed at the time point of 0 lag so that it can provide a more robust

representation of the true measured accuracy of eye-tracking recording. The average accuracy for each group is 1.4410, 0.5835, and 0.8393 for Training set participants, POAG patients, and age-matched healthy controls, respectively (see Figure S2-A and Table S1, first row). These values are in line with those reported by the eye-tracker manufacturer (EyeLink1000 and EyeLink Portable Duo, SR-Research, Ontario, Canada).

The precision of the eye-tracking data is computed by estimating the noise present in the recorded gaze coordinates time-series. Our pre-processing pipeline already filters the time-series using a Butterworth low-pass filter with half-power frequency = 0.5 to remove the high-frequency oscillations present in the data. We then compared the raw signal with the filtered one and calculated the residuals $p(t)_{raw}$ - $p(t)_{filtered}$ (for examples, see Figure S3-A and S3-B). The noise level (and therefore the precision) is estimated as the variance of the distribution of the residuals, which in our case has the shape of a Laplacian distribution (see Figure S3-B). The results are reported in Figure S2-B and Table S1, second row). The average values of precision show that the amount of noise present in the data is negligible across all groups of participants (Training set = 0.0079 POAG = 0.0178, age-matched controls = 0.0051).

The data loss is computed taking into account recording artifacts, gaps in the recording, and participants' blinks. We calculated the ratio between the removed data points and the total length of the time series and that defines the data loss rate. The average data loss does not show significant differences across groups, with average values below 10% (see Figure S2-C and Table S1, third row).

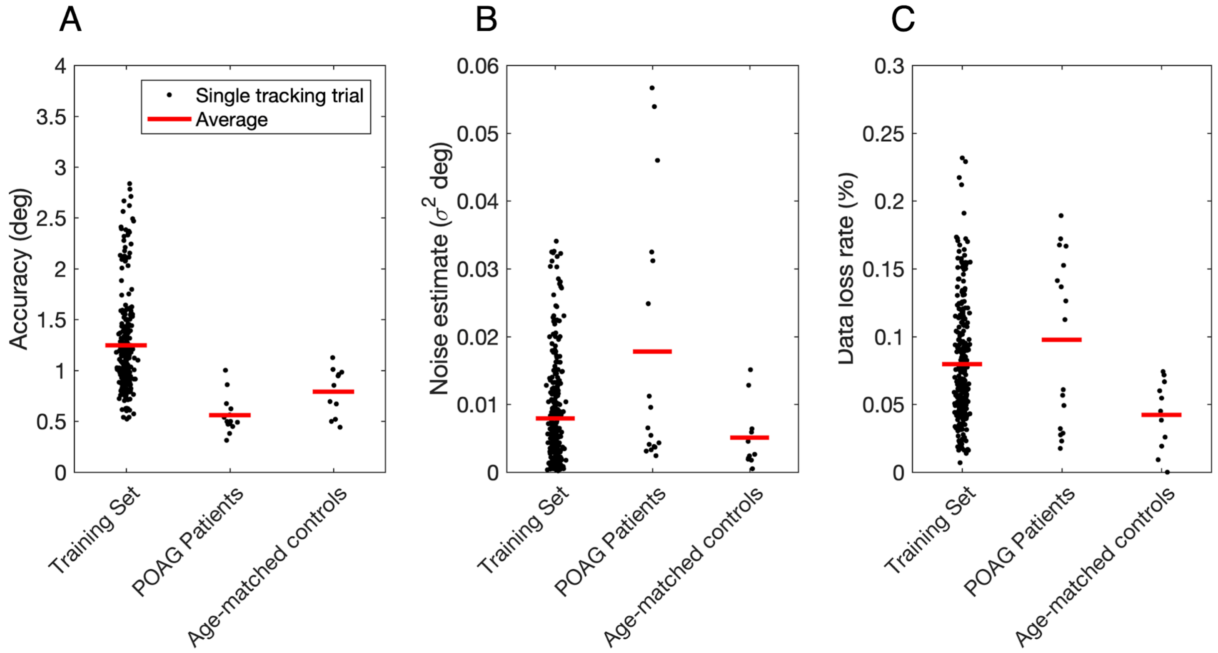

Figure S2. Measures of eye-tracking data quality. **A**. Accuracy, measured as the average absolute error (eye position - stimulus position) at 0 lag. **B**. Precision, measured as the noise present in the recording. It is calculated as the variance of the distribution of residuals between the pre- and post-filtered eye-tracking signal. **C**. Data loss rate, measured including blinks.

	**Training Set**	**POAG patients**	**Age-matched controls**
**Accuracy (deg)**	1.2470 ± 0.4820	0.5600 ± 0.1829	0.7914 ± 0.2364
**Precision (σ^2 deg)**	0.0079 ± 0.0078	0.0178 ± 0.0192	0.0051 ± 0.0048
**Data loss rate (%)**	0.0797 ± 0.0425	0.0976 ± 0.0624	0.0422 ± 0.0258

Table S1. Results of the data quality measures.

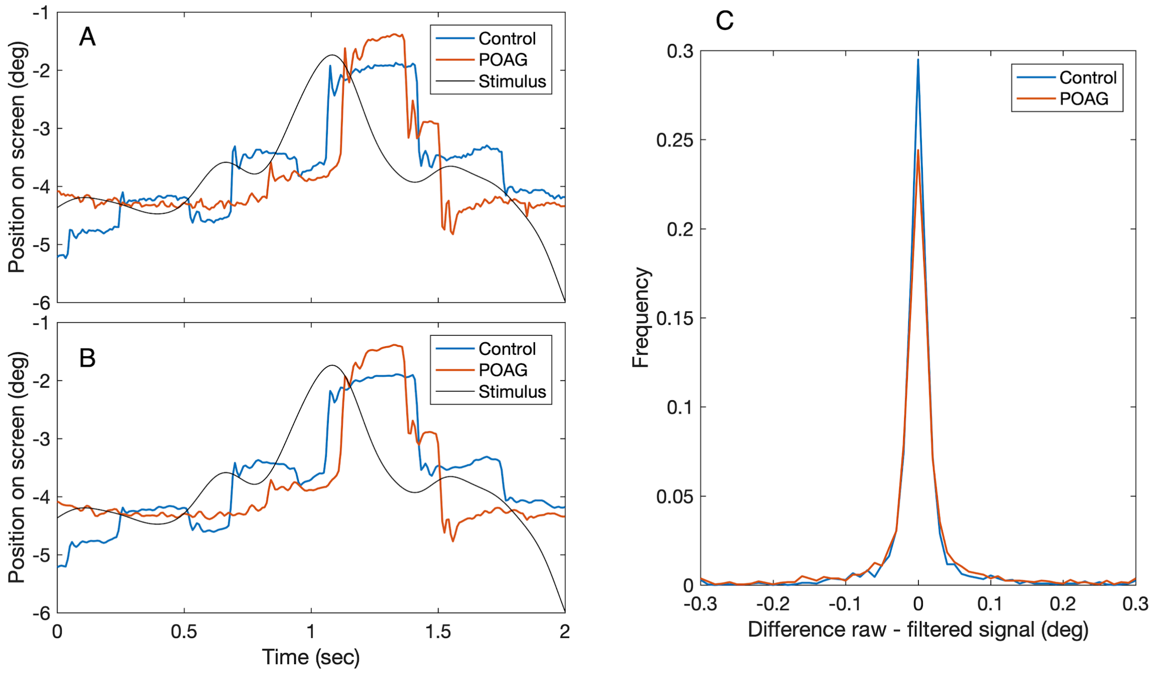

Figure S3. Examples of eye-tracking time-series for a control participant and a POAG patient. **A**. Pre-filtering time-series. **B**. Low-pass filtered time-series. **C**. Distribution of the residuals (A-B). On average, the POAG patients show slightly more noisy signals, though the absolute level of noise is still negligible and easily removed by the filtering during pre-processing.

Testing robustness to miscalibration errors

In a clinical setting, an optimal calibration of the eye-tracking setup is often not feasible due to a patient's conditions or time constraints. Therefore, it is important to develop methods that are as independent as possible from the quality of calibration, or at least robust enough to tolerate a generous margin of error.

We tested how the Recurrent Neural Network performs in classifying visual field defects when the test data is distorted by simulated miscalibrations. This is done by using the output of the upper stream of the network architecture shown in Figure 4, named "Visual Field Classifier". This output is categorical and can indicate one of the 4 classes of visual field defect tested (*no loss*, *central loss*, *peripheral loss*, *hemifield loss*). Noticeably, we did *not* re-train our model for this purpose: as a possible future development, training the RNN with a data-augmentation scheme that introduces miscalibration-like distortions in the training dataset would likely provide increased robustness, as demonstrated in the image data domain (Hernandez-Garcia, König, & Kietzmann, 2019; Rusak et al., 2020).

We tested two types of miscalibration-like distortions: an additive distortion (i.e. offsets) where error constants a and b are added to the horizontal and vertical time-series of gaze positions, as defined in Equation S3:

$$p_{d1}(t) = \left[p_x(t);\ p_y(t)\right] + [a; b];\ (a, b) \in [-5\colon 5\ deg] \tag{S3}$$

And a multiplicative distortion where error coefficients α and β are combined with the horizontal and vertical time-series of gaze positions through element-wise multiplication, as defined in Equation S4:

(S4)

$$p_{d2}(t) = [p_x(t);\ p_y(t)] \odot [\alpha; \beta]; (\alpha, \beta) \in [0.75{:}\ 1.25]$$

Lastly, we tested the combinations of these two types of distortion, as defined in Equation S5:

(S5)

$$p_{d3}(t) = [p_x(t);\ p_y(t)] \odot [\alpha; \beta] + [a; b]$$

The maximum values of a, b, α and β are chosen arbitrarily but they are based on empirical measurements: “normal” miscalibration error can occur in the range of +/-5 deg of offset and +/-25% distortion.

The values of the additive error are chosen by combining horizontal and vertical errors a and b into an absolute radial error $r = \sqrt{a^2 + b^2}$ and then sampling r radially. In this way, we can express the accuracy of classification as a function of the intensity of the distortion expressed as the distance from the original data (e.g. Figure S5-A and S5-B).

See Figure S4-A, where the black dots represent the individual samples and the orange circles are the selected radiuses where the additive error is combined with the multiplicative (Figure S4-C).

The values of the multiplicative error are chosen by combining α and β values in a grid with 0.05 step size (Figure S4-B).

We tested the robustness of the model through 5-fold cross-evaluation, as for the main results of the paper, and found that the categorical classification of the RNN is remarkably robust to different types of miscalibration errors. For additive-only errors (Figure S4-A and S5-B) the classification accuracy stays above chance level (which in our case is 25%, given the four types of possible visual field defect) for the whole range of distortions tested. Noticeably, up until almost 2 degrees the RNN performance is practically unaffected, with an accuracy stably above 90%. When subjected to multiplicative-only errors (Figure S4-B) the RNN well-tolerates a +15% / -10%

distortion along the horizontal axis and a ±25% distortion along the vertical axis. We speculate that horizontal-vertical difference is due to the aspect ratio of the monitor used for the data collection, and hence the range of possible movements of the stimulus. The vertical axis of the trajectory was always contained in approximately 20 degrees of visual angle, while the horizontal axis spanned over almost 40 degrees of visual angle.

When combining additive and multiplicative errors (Figure S4-C and S5-A) we found that the drop in accuracy is driven primarily by both horizontal multiplicative and additive errors above 1 degree.

Finally, it is important to note that all these errors were added on top of the actual data recordings, which inevitably also carry their own miscalibration and that have an average deviation from the true signal of 1.25 degrees, with it reaching 3 degrees in some participants (see Figure S2-A). This means that the RNN, when performing visual field defect classification, can reliably tolerate total deviations of around ±4 degrees, and still perform above chance with a hypothetical calibration error of ±8 degrees.

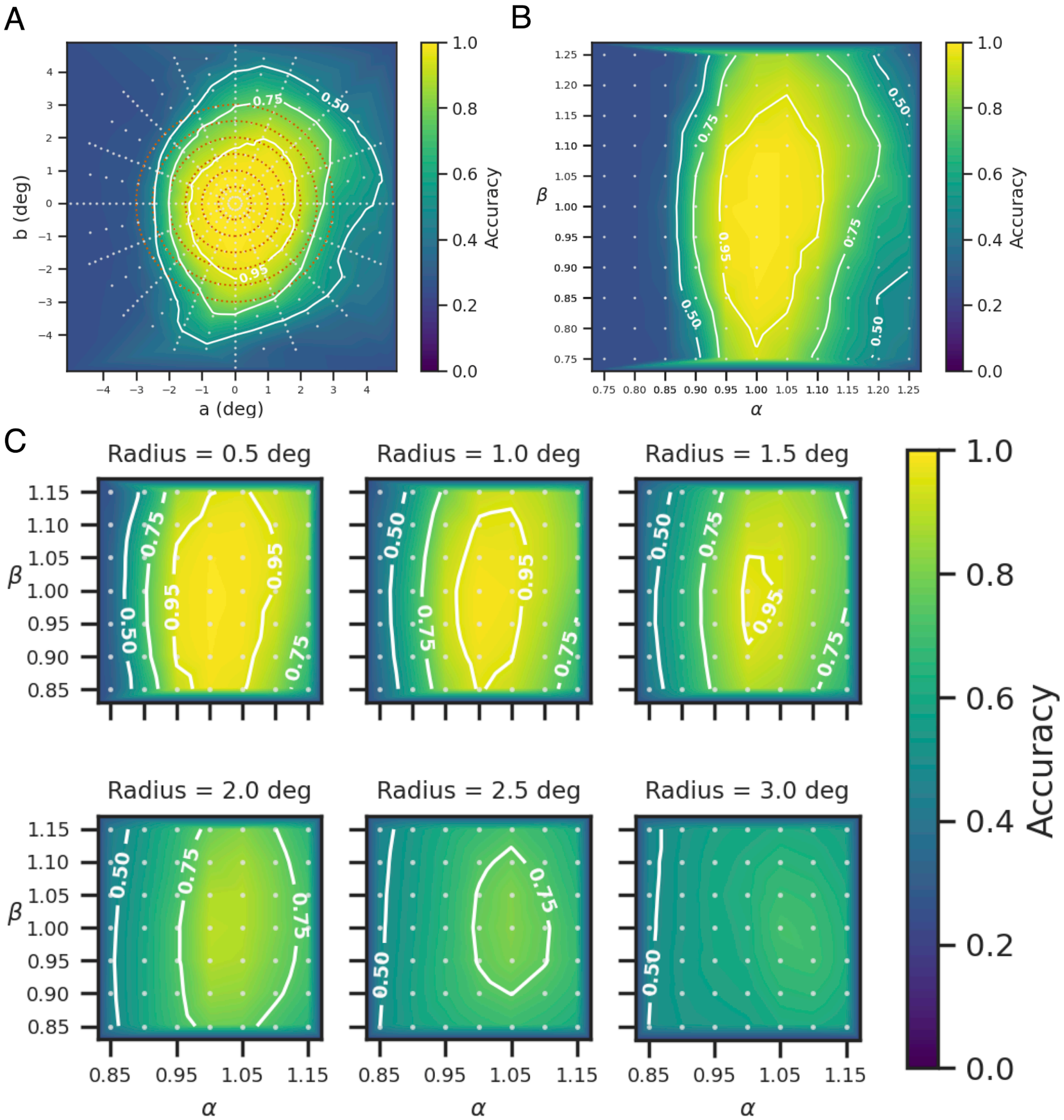

Figure S4. Mean accuracy of the RNN performing visual field defect classification into 4 classes (no loss, central loss, peripheral loss, hemifield loss) in the presence of calibration additive-only errors (**A**), multiplicative-only errors (**B**) and combined additive-multiplicative errors (**C**). The gray dots represent the combination of a and b or α and β values tested and the values between samples were linearly interpolated. The orange circles in panel A are the radiuses tested for the combined additive-multiplicative errors in panel C.

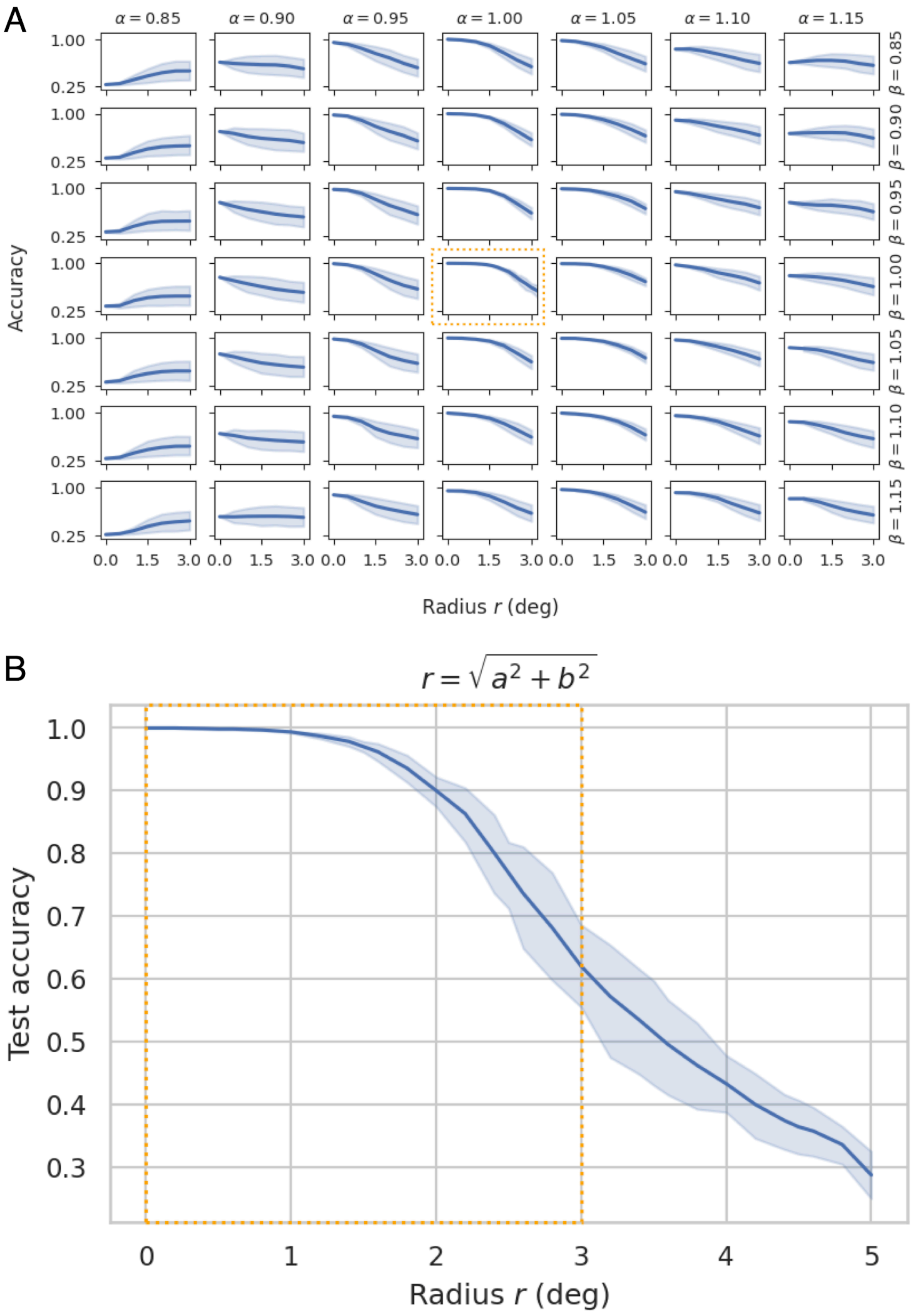

Figure S5. Accuracy of the RNN in classifying the visual field defects as a function of the absolute radial error. **A.** All combinations of additive and multiplicative errors. **B.** Detail of the classification accuracy when additive-only errors are introduced to the data.

References

Akaike, H. (1969). Fitting autoregressive models for prediction. *Annals of the Institute of Statistical Mathematics*, Vol. 21, pp. 243–247.

Hernandez-Garcia, A., König, P., & Kietzmann, T. (2019). Learning robust visual representations using data augmentation invariance. *2019 Conference on Cognitive Computational Neuroscience*. doi:10.32470/ccn.2019.1242-0

Rusak, E., Schott, L., Zimmermann, R. S., Bitterwolf, J., Bringmann, O., Bethge, M., & Brendel, W. (2020). A simple way to make neural networks robust against diverse image corruptions. *ArXiv Preprint ArXiv:2001. 06057*. Retrieved from https://www.ecva.net/papers/eccv_2020/papers_ECCV/papers/123480052.pdf
